# The social psychological impact of the COVID-19 pandemic on medical staff in China: A cross-sectional study

**DOI:** 10.1192/j.eurpsy.2020.59

**Published:** 2020-06-01

**Authors:** Zai-Quan Dong, Jing Ma, Yan-Ni Hao, Xiao-Ling Shen, Fang Liu, Yuan Gao, Lan Zhang

**Affiliations:** 1 Mental Health Center of West China Hospital, Sichuan University, Chengdu, China; 2 Department of Medical Affairs, West China Hospital, Sichuan University, Chengdu, China; 3 Information Technology Center of West China Hospital, Sichuan University, Chengdu, China

**Keywords:** COVID-19, cross-sectional survey, novel coronavirus, medical staff, psychology

## Abstract

**Background.:**

The COVID-19 outbreak required the significantly increased working time and intensity for health professionals in China, which may cause stress signs.

**Methods.:**

From March 2–13 of 2020, 4,618 health professionals in China were included in an anonymous, self-rated online survey regarding their concerns on exposure to the COVID-19 outbreak. The questionnaires consisted of five parts: basic demographic information and epidemiological exposure; occupational and psychological impact; concerns during the episode; coping strategies; and the Huaxi Emotional-Distress Index (HEI).

**Results.:**

About 24.2% of respondents experienced high levels of anxiety or/and depressive symptoms since the COVID-19 outbreak. Respondents who worried about their physical health and those who had COVID-19 infected friends or close relatives were more likely to have high HEI levels, than those without these characteristics. Further, family relationship was found to have an independent protective effect against high HEI levels. Their main concerns were that their families would not be cared for and that they would not be able to work properly. Compared to respondents with clear emotional problems, those with somewhat hidden emotional issues adopted more positive coping measures.

**Conclusions.:**

About a quarter of medical staff experienced psychological problems during the pandemic of COVID-19. The psychological impact of stressful events was related to worrying about their physical health, having close COVID-19 infected acquaintances and family relationship issues. Therefore, the psychological supprot for medical staff fighting in the COVID-19 pandemic may be needed.

## Introduction

The pneumonia pandemic caused by the 2019 novel coronavirus has rapidly spread from Wuhan to other regions of the world [1]. The World Health Organization (WHO) has declared the coronavirus disease 2019 (COVID-19) as a Public Health Emergency of International Concern (PHEIC) [2]. Moreover, on March 13, 2020, the confirmed infections in China had reached 80,813 [3]. All these evidences indicate that this disease is more dangerous than severe acute respiratory syndrome (SARS) was in 2003 [4]. The COVID-19 outbreak has created considerable panic and, due to its rapid spread, the healthcare system is under unprecedented strain. Because of long shifts and high-intensity work, medical staff are experiencing great stress and thus are at high risk of infection.

While patients need psychological support in clinical treatment, medical staff does as well. During the SARS epidemic, anxiety and fear were common in front-line workers [5]. As learned from Ebola cases, the absence of mental health and psychosocial support systems increases the risks of psychological distress and progression toward psychopathology in medical staff [6]. A higher prevalence of psychological symptoms was found among medical health workers during COVID-19 than in previous pandemics and epidemics [7]. According to one study [8], the prevalence of depression in health professionals reached 50.7%, and stress-related symptoms reached 70.4%. Lai et al. [9] reported that a considerable proportion of healthcare workers had symptoms of depression (50.4%), anxiety (44.6%), insomnia (34.0%), and distress (71.5%). Zhang et al. [10] compared 927 medical health workers with nonmedical health workers and found that medical health workers had a higher prevalence of insomnia, anxiety, depression, somatization, and obsessive–compulsive symptoms. Huang et al. [11] also discovered high incidence of anxiety (23.04%) and stress disorder (27.39%) in first-line medical workers. Our study examines the emotional states, psychosocial factors, and coping strategies of medical staff during the COVID-19 pandemic in order to provide a basis for psychological intervention and other types of support for this group.

## Methods

Using a convenience sampling method, we invited all staff members from 33 hospitals in Sichuan and Yunnan provinces to participate in a cross-sectional survey. Given the intense schedule of front-line workers, we wanted to investigate the data of second-line medical workers. The study covered the period from March 2 to 13, 2020, a relatively stable phase of the pandemic in China. Data were collected through an anonymous, self-rated questionnaire over the Internet (to which all hospital workers had free access). The authors assert that all procedures contributing to this work comply with the ethical standards of the relevant national and institutional committees on human experimentation and with the Helsinki Declaration of 1975, as revised in 2008. All procedures involving human subjects/patients were approved by the Ethics Committee of West China Hospital of Sichuan University. Verbal informed consent was obtained from all subjects, witnessed, and formally recorded.

The questionnaire consisted of five parts: basic demographic information and exposure to the COVID-19 outbreak; occupational and psychological impact; concerns during the episode; coping strategies; and the Huaxi Emotional-Distress Index. Each person could answer the questionnaire only once.

### Basic demographic information and exposure to the COVID-19 outbreak

Basic demographic information included age, gender, educational level, years in practice, marital status, monthly family income, and number of cohabitants. Respondents answered questions about their exposures to COVID-19 during the outbreak, including contact with people from the Wuhan area in the past 2 weeks (“Wuhan exposure”), whether someone had been diagnosed COVID-19 in their community in the past 2 weeks (“community exposure”), whether they had treated patients who developed COVID-19 (“COVID-19 patient”), and whether they had a friend or close relative who developed COVID-19 (“COVID-19 acquaintance”).

Other factors were included related to epidemiological exposure, such as profession, hospital rank (from general to tertiary), hospital category (added after presurvey), and permanence in work.

### Occupational and psychological impact

Six questionnaire items were used to assess the perception of medical staff regarding the occupational and psychological impact of the pandemic. The items were adapted from those used in a previous study assessing the psychological impact of SARS in hospital employees in Canada [12]. Three of these items addressed perceptions of occupational impact during the outbreak:

1. How do you view the influence of COVID-19 on your career? (“I have strengthened my belief as a medical worker”; “Not affected”; and “I will reconsider whether to continue in the medical industry”).
2. What do you think is your most urgent need after the outbreak of COVID-19? (“Choose one of the following: income increase, improved medical condition, more psychological support, or decreased the demand for title promotion”).
3. How do you characterize your attitude towards participating in frontline work (“Chosen”; “Does not matter”; “Unwilling”).

Three other items addressed staff’s perceptions of the psychological impact of the situation:

1. Emotional control during the COVID-19 outbreak (“Hard” or “Easy”).
2. Dreams related to COVID-19 recently (“Often” or “Never”).
3. Perceived risk: In general, how worried you are about the risk of COVID-19 to your current life? (“Very worried” or “Not too much”).

### COVID-19 concerns and coping methods

Based on previous research [12], 14 questions were designed to investigate the respondents’ concerns, and other 10 questions were designed to investigate what strategies they used to address the COVID-19 threat.

### Huaxi Emotional-Distress Index (HEI)

The HEI was used to screen emotional distress (anxiety, depression, and/or suicidal ideation) in medical staff during the last month. The HEI includes nine self-reported items that can be finished in less than 5 min. A total score of >8 points indicates that the respondent has clear negative emotions and related mental health problems. The Cronbach’s α of HEI was 0.90 (0.915 in this study); sensitivity and specificity were 0.880 and 0.766, respectively [13].

### Statistical analysis

All statistical analyses were performed using SPSS 22.0. The Chi-square test, Fisher’s exact probability method, and *t* test (for continuous variables) were used to identify potential predictive and associated factors. A multiple logistic regression analysis (stepwise forward) was performed by including variables based on the inclusion criteria. Logistic regression analyses were subsequently conducted in four steps, with the outcome variable being a high level of HEI score. In Model 1, variables measuring exposure to the COVID-19 outbreak (Wuhan exposure, community exposure, COVID-19 patient, and COVID-19 acquaintance) and variables considered related to work exposure (including hospital category, hospital rank, profession, and return to work or not) were entered into the equation. In Model 2, “feelings about health condition” was added into the model. In Models 3 and 4, perceived risk and family relationship were added in respectively. Variables of gender, age, education, marriage, income, and number of cohabitants were controlled in all steps during the logistic regression analyses.

## Results

### Descriptive and bivariate analyses

The first column of Table 1 shows the characteristics of the total sample. A total of 4,618 questionnaires were completed online. Respondents comprised 3,863 (86.7%) women and 755 (16.3%) men, 41.3% between 30 and 39 years old, 16.8% between 40 and 49 years old, and 6.9% who were 50 years or older. Most of the respondents were nurses (*n* = 2,889, 62.6%) and doctors (*n* = 1,138, 24.6%); the rest were technicians (*n* = 319, 6.9%) and health administrators (*n* = 272, 5.9%). Their length of work experience varied from less than 1 year to more than 50 years, with an average of 12.19 (standard deviation [SD] = 9.39) years. Most of them were married (*n* = 3,509, 76.0%) and 3,899 (84.4%) health professionals returned from holiday to work after the outbreak. Also, 859 (19.5%) respondents admitted to having a history of epidemiological exposure.

**Table 1.** Bivariate association between level of HEI score and related factors.

Variables		Total, *n* (%)	HEI ≤ 8, *n* (%)	HEI > 8, *n* (%)	*p* value
Gender (*n* = 4,618)	Male	755 (16.3)	605 (17.3)	150 (13.4)	0.002^a^
	Female	3,863 (83.7)	2,895 (82.7)	968 (86.6)	
Age，years (*n* = 4,618)	≤29	1,617 (35.0)	1,210 (34.6)	407 (36.4)	0.366^a^
	30–39	1,905 (41.3)	1,438 (41.1)	467 (41.8)	
	40–49	777 (16.8)	604 (17.3)	173 (15.5)	
	≥50	319 (6.9)	248 (7.1)	71 (6.4)	
Years in practice (*n* = 4,618, *M*[SD])		12.19 (9.39)	12.30 (9.50)	11.86 (9.07)	0.184^b^
Educational level (*n* = 4,618)	≤High school	128 (2.8)	98 (2.8)	30 (2.7)	0.836^a^
	>High school	4,490 (97.2)	3,402 (97.2)	1,088 (97.3)	
Marital status	Married	3,509 (76.0)	2,670 (76.3)	839 (75.0)	0.695^a^
	Single	968 (21.0)	725 (20.7)	243 (21.7)	
	Divorced or widowed	141 (3.1)	105 (3.0)	36 (3.2)	
Number of cohabitants (*n* = 4,618, *M*[SD])		3.72 (1.83)	3.71 (1.84)	3.75 (1.79)	0.604^b^
Monthly household income, (*n* = 4,618)	<10,000	2,918 (63.2)	2,167 (61.9)	751 (67.2)	0.002^a^
	≥10,000	1,700 (36.8)	1,333 (38.1)	367 (32.8)	
Profession (*n* = 4,618)	Doctor	1,138 (24.6)	868 (24.8)	270 (24.2)	0.006^a^
	Nurse	2,889 (62.6)	2,152 (61.5)	737 (65.9)	
	Technician	319 (6.9)	258 (7.4)	61 (5.5)	
	Health administrators	272 (5.9)	222 (6.3)	50 (4.5)	
Hospital category (*n* = 4,194)	Specialized hospital	568 (13.5)	444 (14.1)	124 (11.8)	0.062^a^
	General hospital	3,626 (86.5)	2,702 (85.9)	924 (88.2)	
Hospital rank (*n* = 4,618)	Tertiary hospital	2,725 (59.0)	2,080 (59.4)	645 (57.7)	0.494^a^
	Second-class hospital	1,640 (35.5)	1,234 (35.3)	406 (36.3)	
	Primary hospital	253 (5.5)	186 (5.3)	67 (6.0)	
Feelings about physical health condition (*n* = 4,618)	Good	3,658 (79.2)	2,952 (84.3)	706 (63.1)	<0.001^a^
	Not so good	960 (20.8)	548 (15.7)	412 (36.9)	
Return to work (*n* = 4,618)	Yes	3,899 (84.4)	2,952 (84.3)	947 (84.7)	0.771^a^
	No	719 (15.6)	548 (15.7)	171 (15.3)	
Perceived risk (*n* = 4,618)	Very worried	3,380 (73.2)	2,384 (68.1)	996 (89.1)	<0.00^a^
	Not too much	1,238 (26.8)	1,116 (31.9)	122 (10.9)	
Family Relationships (*n* = 4,618)	Good	4,252 (92.1)	3,289 (94.0)	963 (86.1)	<0.001^a^
	Not so good	366 (7.9)	211 (6.0)	155 (13.9)	
Wuhan exposure (*n* = 4,618)	No	4,483 (97.1)	3,410 (97.4)	1,073 (96.0)	0.012^a^
	Yes	135 (2.9)	90 (2.6)	45 (4.0)	
Community exposure (n = 4,618)	No	4,062 (88.0)	3,084 (88.1)	978 (87.5)	0.569^a^
	Yes	556 (12.0)	416 (11.9)	140 (12.5)	
COVID-19 patient (*n* = 4,618)	No	4,202 (91.0)	3,213 (91.8)	989 (88.5)	0.001^a^
	Yes	416 (9.0)	287 (8.2)	129 (11.5)	
COVID-19 acquaintance (*n* = 4,618)	No	4,419 (95.7)	3,380 (96.6)	1,039 (92.9)	<0.001^a^
	Yes	199 (4.3)	120 (3.4)	79 (7.1)	

Abbreviation: HEI: Huaxi Emotional-Distress Index; SD, standard deviation.

a Chi-square test was used.

b Two-sided independent sample *t* test was used.

The HEI scores in this sample ranged from 0 to 36, with a mean of 5.49. About 24.2% (*n* = 1,118) of the employees reported having high levels of mental health issues (i.e., a HEI score of 9 or more), including 14.9% (*n* = 688) with mild negative emotions (HEI score of 9–12), 5.5% (*n* = 254) with moderate negative emotions (HEI scores of 13–16) and 3.8% (*n* = 176) with severe negative emotions (HEI score of 17 or more), respectively. The results of the bivariate analysis (Table 1) indicated that among sociodemographic factors, high HEI score was associated with women, low monthly household income, nurses, negative feelings about physical health condition, bad family relationships, and having epidemiological exposure.

### Factors related to high HEI levels

To further elucidate the relation among outbreak event exposures, risk perception, family relationships, and level of HEI score, logistic regression analyses were conducted (Table 2). In Model 1, after controlling for variates with significant differences, gender and income (*p* = 0.018, 0.022, respectively), and variates with no significant differences, such as age, education, marital status, and number of cohabitants (all *p* > 0.05), acquaintance exposure variables retained their significant relations with high HEI levels, with an adjusted odds ratio of 2.122 (*p* < 0.001). The adjusted odds ratio of technicians was 0.719 compared to doctors (*p* = 0.046). In Model 2, when “feelings about health condition” was added into the regression equation, the associations between higher HEI score and profession diminished, suggesting that this variable may partially mediate the effects of direct outbreak exposure on HEI levels. However, the impact of COVID-19 acquaintance remained significant in Model 2. In Model 3, when perceived risk was added, the association between higher HEI score and high perceived risk was found. Finally, in Model 4, when family relationship was added, an independent protective effect against high HEI levels was shown.

**Table 2.** Logistic regression analysis of factors associated with high HEI score.^a^

Factors	Model 1		Model 2		Model 3		Model 4		
	*p*	OR (95% CI)	*p*	OR (95% CI)	*p*	OR (95% CI)	*p*	OR (95% CI)	
Hospital category (specialized hospital)	0.164	0.856 (0.688–1.065)	0.213	0.867 (0.693–1.085)	0.186	0.857 (0.683–1.077)	0.135	0.84 (0.668–1.056)	
Profession (doctor)	NA	0 (Reference)	NA	0 (Reference)	NA	0 (Reference)	NA	0 (Reference)	
Profession (nurse)	0.958	1.005 (0.827–1.221)	0.502	1.071 (0.877–1.307)	0.316	1.11 (0.905–1.361)	0.245	1.129 (0.92–1.386)	
Profession (technician)	0.046	0.719 (0.520–0.993)	0.142	0.781 (0.561–1.086)	0.106	0.758 (0.542–1.060)	0.115	0.763 (0.545–1.068)	
Profession (health administrative)	0.051	0.691 (0.477–1.002)	0.178	0.771 (0.529–1.125)	0.351	0.833 (0.567–1.223)	0.342	0.83 (0.564–1.22)	
Hospital rank (tertiary hospital)	NA	0 (Reference)	NA	0 (Reference)	NA	0 (Reference)	NA	0 (Reference)	
Hospital rank (second-class hospital)	0.284	0.831 (0.593–1.166)	0.356	0.849 (0.600–1.202)	0.556	0.899 (0.629–1.283)	0.704	0.933 (0.652–1.335)	
Hospital rank (primary hospital)	0.305	0.84 (0.602–1.172)	0.498	0.888 (0.630–1.251)	0.452	0.874 (0.616–1.241)	0.549	0.898 (0.630–1.278)	
Wuhan exposure (yes)	0.109	1.376 (0.931–2.032)	0.145	1.347 (0.902–2.011)	0.139	1.362 (0.905–2.051)	0.202	1.308 (0.865–1.977)	
Community exposure (yes)	0.764	0.967 (0.775–1.206)	0.581	0.938 (0.748–1.177)	0.503	0.924 (0.734–1.164)	0.494	0.922 (0.732–1.162)	
COVID-19 acquaintance (yes)	0.000	2.122 (1.539–2.925)	0.000	2.102 (1.511–2.925)	0.000	1.899 (1.357–2.656)	0.000	1.872 (1.335–2.624)
COVID-19 patient (yes)	0.078	1.239 (0.976–1.573)	0.120	1.215 (0.951–1.552)	0.189	1.182 (0.921–1.516)	0.161	1.196 (0.931–1.536)
Return to work (yes)	0.817	1.023 (0.841–1.245)	0.975	0.997 (0.815–1.219)	0.975	0.997 (0.812–1.223)	0.960	1.005 (0.818–1.235)
Feeling about physical health condition (good)	NA	NA	0.000	0.323 (0.275–0.379)	0.000	0.358 (0.304–0.421)	0.000	0.395 (0.334–0.469)
Perceived risk (worried)	NA	NA	NA	NA	0.000	3.571 (2.888–4.417)	0.000	3.605 (2.912–4.462)
Family relationships (good)	NA	NA	NA	NA	NA	NA	0.000	0.551 (0.427–0.71)

Abbreviations: CI, confidence intervals; HEI, Huaxi Emotional-Distress Index; NA, not applicable; OR, odds ratio.

a In all models, gender, age, education, marital status, income and number of cohabitants were controlled for.

### The occupational and psychological impact of the COVID-19 outbreak

A total of 2,842 (61.5%) respondents reported that COVID-19 strengthened their determination in being a health professional, and only 264 respondents (5.7%) reported that the outbreaks had caused them to re-evaluate their career choice. Despite these findings, most respondents (*n* = 4,120, 89.2%) strongly wanted to participate in frontline work. The most urgent need for medical staff was to increase their income (*n* = 2,098, 45.4%) and improve work conditions (*n* = 1,579, 34.2%); 10.3% (*n* = 477) chose psychological support as their most urgent need. Further, 14.2% (*n* = 655) reported that it was hard for them to control their emotions during the COVID-19 outbreak, and 30.6% (*n* = 1,411) had recently dreamed about COVID-19.

### COVID-19 related concerns

Among the scenarios listed in Table 3, the areas of greatest concern for health professionals included “Families are not protected because of the lack of protective material (masks, etc.),” “I will not be able to care for loved ones,” “Non-COVID patient care will lose quality care,” and “I will not be able to work.”

**Table 3.** COVID-19 related concerns among medical staff.^a^

Concern	Mean rating ± SD
Non-COVID patient care will lose quality	2.81 ± 1.06
I will not be able to travel	1.94 ± 1.06
I will spread COVID to living companions	2.45 ± 1.31
I will get COVID from touching objects in hospital	2.69 ± 1.09
I will not be able to care for loved ones	2.88 ± 1.20
I will not be able to enjoy my usual social activities	2.33 ± 1.10
I will not be able to work	2.83 ± 1.21
I will get COVID from the air I breathe	2.25 ± 1.08
I will spread COVID to others in public	2.26 ± 1.18
My education or teaching will be interrupted	2.24 ± 1.13
I will get very sick or die from COVID	1.97 ± 1.07
I am afraid I will be discriminated against, or not touched, because I work in the hospital	2.01 ± 1.07
Families are not protected because of the lack of protective material (masks, etc.)	2.98 ± 1.21
Travel inconvenience	2.56 ± 1.54
My child’s study plan was disrupted	2.58 ± 1.40

Abbreviation: SD, standard deviation.

a Based on a 5-point Likert-type scale: 1, not concerned; 5, extremely concerned.

### Coping strategies used by medical staff to address the COVID-19 emergency

Medical staff coping methods regarding COVID-19 are presented in Table 4. As shown, medical staff without emotional problems were significantly more likely to cope by “adhering to infection control procedures,” “just accepting the risks,” “keeping a positive mindset,” “keeping a healthy lifestyle,” “avoiding thinking about the risks,” “avoiding traveling,” and less “taking vitamins, herbs, or other complementary substances” than respondents with obvious emotional problems.

**Table 4.** COVID-19 coping strategies among medical staffs in China.

Coping strategy	Chosen	Total (*n* = 4,618)	HEI ≤ 8 (*n* = 3,500)	HEI > 9 (*n* = 1,118)	*p* value
Adhering to infection control procedures	Yes	4,594	3,486	1,108	0.045^a^
	No	24	14	10	
Staying informed about COVID-19	Yes	4,605	3,494	1,114	0.591^b^
	No	13	9	4	
Just accepting the risks	Yes	4,320	3,305	1,015	<0.001^a^
	No	298	195	103	
Keeping a positive mindset	Yes	4,589	3,488	1,101	<0.001^a^
	No	29	12	17	
Keeping a healthy lifestyle	Yes	4,585	3,486	1,099	<0.001^a^
	No	33	14	19	
Talking to others	Yes	3,708	2,827	881	0.149^a^
	No	910	673	237	
Avoiding crowds or people with colds	Yes	4,455	3,372	1,083	0.406^a^
	No	163	128	35	
Avoiding thinking about the risks	Yes	1,195	951	244	<0.001^a^
	No	3,423	2,549	874	
Avoiding traveling	Yes	4,404	3,364	1,043	<0.001^a^
	No	211	136	75	
Taking vitamins, herbs, or other complementary substances	Yes	2,006	1,459	547	<0.001^a^
	No	2,612	2,041	571	

Abbreviations: HEI, Huaxi Emotional-Distress Index; No, this method was rarely used; Yes, this method was often used.

a Chi-square test was used.

b Fisher’s exact probability method was used.

## Discussion

In any situation in which every measure of prevention and control is important, there are serious public psychological costs [14]. For health professionals, this pandemic has been a blow to their work and their personal lives. The COVID-19 outbreak has brought feelings of loss of control, uncertainty, and vulnerability [5].

The present study suggests that 24.2% of medical staff reported high levels of psychological issues, including anxiety and depressive emotion, sometimes severe. Our result is lower than some studies’, including Lai et al.’s on front-line medical staff [5, 7, 9], but similar to other recent studies in China [10, 11]. This difference may be due to different survey instruments used and different timing of surveys.

Groups exposed to pandemics tend to be mentally fragile when under distress. Similar results were seen in a cross-sectional study of rescue workers exposed to radiation after the Great East Japan Earthquake, where the prevalence of probable severe mental illness reached 21.4% [15]. The impact of exposure history has also been confirmed by several surveys [16, 17]. Our findings show that having a friend or close relative who developed COVID-19 was the only relative factor contributing to a high HEI score, consistent with the literature on SARS [16]. In addition, perception of bodily symptoms independently contributed to high HEI scores and revealed the interaction between body and mind. Family relationships were shown to be a protective factor against high HEI score. The loss of contact with relatives results in physical and psychological isolation [18] and can put stress on relationships; favorable relationships act as social support in crises [19]. Thus, during the period of the pandemic, medical staff who have the above risk factors require special attention.

Previous research suggests that many clinical workers experience professional distress in widespread disasters [5, 12], which is unsurprising, given their exposure risk and long, intense shift work. Our study confirmed, however, that despite high stress, medical staff in China mostly expressed positive feelings for their professions. This unprecedented bio-disaster never seem to sway their belief; instead, they showed courage and commitment to their occupation.

The most worrisome problem for medical staff, as might be expected, was related to their loved ones. Given the transmission characteristics of COVID-19, working at high risk of infection made people afraid of passing the virus to their family and friends [12, 20–22]. Shortages of masks and other necessities, tense relationships with their children due to the quarantine, and canceling of normal family activities probably contributed to medical staff’s feelings of insecurity. Furthermore, when they see their colleagues rush to the center of the epidemic [23], infected employees feel guilty for not working, as their professional responsibility and energy drive them to do. As several studies have confirmed, loss of income during this time is also frightening [12], and disaster responders may experience greater psychological problems postincident if they suffer property loss [24,25]. For that reason, the Chinese government has decided to raise the salaries of health professionals [26].

According to our results, the group who had lower HEI scores mostly chose a different way to express emotions, perhaps because of different coping methods. Health care workers’ stress tends to disturb their emotions and weaken their coping behavior. For instance, being quarantined is significantly and positively associated with avoidance behavior [5]. Further, interpersonal communication hindered by N95 masks and protection suits may induce bad tempers or suppressed emotions. Favorable social support and response strategies are essential for reducing stress provisionally as well as lowering risk of long-lasting effects [27,28]. Thus, applying positive coping strategies during this hard time is fundamentally important. Accordingly, the coping strategies that most healthy participants adopted should be emphasized:

1. Comply with infection control procedures. This reduces the risk of infection and also reduces corresponding psychological stress.
2. Accept risks and avoid thinking about them. Try to take it easy.
3. Keep a positive mindset.
4. Maintain a healthy lifestyle, get enough sleep, and exercise.
5. Avoid, or at least reduce, traveling.
6. Use vitamins, herbs, and other complementary substances with caution when their effects are uncertain.

In addition, as mentioned in a previous survey [21, 29], rest times and places for medical staff are essential, so a rest schedule [30] and several resting areas should be established. It is also urgent to arrange other related forms of government financial support and psychological assistance.

This study has several limitations. First, this survey was unable to sample front-line clinical workers sufficiently, those who treated COVID-19 patients directly. That group might have more mental health issues [9,19,31,32]. Second, we were limited to the online mode because the virus hinders face to face communication; the online anonymous questionnaire was the safest data-collecting choice. Third, convenience sampling might have affected the representativeness of sample (although in this tense situation, a better solution remains to be discovered). Fourth, we used short, quick tools to assess the mental state of participants, which had an advantage in speed but a disadvantage in consistency assessment. Fifth, this study did not include some other anxiety-related symptoms and possible psychological variables, such as the post-traumatic stress [33] common to healthcare workers. Consequently, further research could expand the coverage and diversity of sample and add layers in study design.

Health-care workers are at high risk of mental issues in this crisis. Our study, targeted on this special population, provides an appropriate way to learn more about their needs and could be a reference for further and more powerful policies. The COVID-19 pandemic provides a lesson in improved mental health and psychosocial support systems in China, such as the provision of online mental services during this hard time. After the crisis, the transformation of mental assistance from short- to long-term is expected. We believe that powerful governmental action can strengthen public faith in conquering this pandemic as well as reducing the distress it causes.

## Data Availability

We do not make our resources publicly available.
